# A model for training medical student innovators: the Harvard Medical School Center for Primary Care Abundance Agents of Change program

**DOI:** 10.3402/meo.v21.30662

**Published:** 2016-06-14

**Authors:** David B. Duong, Erin E. Sullivan, Myechia Minter-Jordan, Lindsay Giesen, Andrew L. Ellner

**Affiliations:** 1 Department of Internal Medicine, Brigham & Women's Hospital, Boston, MA, USA; 2 Harvard Medical School Center for Primary Care, Boston, MA, USA; 3 Dimock Health Center, Boston, MA, USA; 4 RTI International, San Francisco, CA, USA; 5 Program in Global Primary Care and Social Change, Harvard Medical School, Boston, MA, USA

**Keywords:** leadership, innovation, community health centers, interprofessional, teams

## Abstract

**Background:**

In 2013, the Harvard Medical School Center for Primary Care established the Abundance Agents of Change (AoC) program to promote interprofessional learning and innovation, increase partnership between 15 academic and community health centers (CHCs) in Boston's most under-served communities, and increase medical student interest in primary care careers.

**Methods:**

The AoC is modeled in the form of a ‘grants challenge’, offering $20,000 to interprofessional student teams to develop an innovative solution that addresses a healthcare delivery need identified by CHCs. The program's initial two years were characterized by a four-stage process which included working with CHCs and crafting a request for proposals, forming interprofessional 20 student teams comprising students from across and outside of Harvard University, training students using a systems-based innovation curriculum, and performing program evaluation.

**Results:**

Our evaluation data from cohorts 1 and 2 of the AoC program demonstrate that we succeeded in training students as innovators and members of interprofessional teams. We also learned valuable lessons regarding creating better alignment with CHC priorities, extending the program cycle from 12 to 18 months, and changing the way funding is disbursed to 25 students, which will be incorporated in later versions of the program.

**Conclusions:**

Based on our experience and evaluation data, we believe that this program is a replicable way to train students as innovators and members of interprofessional teams to address the current complex healthcare environment.

Current healthcare delivery systems face multi-dimensional problems that require solutions spanning political, social, biomedical, and technological disciplines. Improving these systems will require interprofessional collaboration and engaging stakeholders to identify innovative solutions to entrenched problems (1). Experts have identified systems-based learning as the next major paradigm shift in healthcare education (2). The Accreditation Council for Graduate Medical Education (ACGME) and the American Board of Medical Specialties (ABMS) define systems-based practice as a core competency that requires students to learn how patient care relates to the healthcare system and how healthcare systems can improve the quality and safety of care (3, 4). This re-orientation of medical training toward a broader health systems approach will require students to gain a more integrated view of healthcare delivery focused on public health, communities, and other factors directly impacting the social determinants of health (5).

In 2013, the Center for Primary Care at Harvard Medical School received a philanthropic grant to design and implement the Abundance Agents of Change (AoC) program. The AoC set out to achieve the following aims: 1) promote interprofessional learning, leadership, and teamwork; 2) foster innovative approaches to serving Boston's most underserved communities; and 3) increase medical student interest in primary care careers. The program catalyzed collaboration among students from within and outside of health-related disciplines (e.g., law, engineering, policy, or business); assigned the teams to physician-mentors at four community health centers (CHCs); and charged each team to identify a problem, implement a system-based solution, and measure its success. In this study, we review the literature describing other programs addressing similar goals, describe the formation and implementation of AoC, and report the results of an initial evaluation.

## Methods

### Literature review

We sought examples in the published literature that could potentially serve as models for AoC. We searched multiple databases (PubMed, Web of Science, ERIC, Science Direct, CINAHL, PsycINFO and EMBASE) from January 2000 through December 2014 to identify reports of programs focusing on training interprofessional teams how to innovate in order to solve healthcare problems. We used the following search terms and their variants, in all possible combinations: medical student, curriculum, interprofessional, team, innovation, and community. All papers were separately and individually assessed. Cross-listed papers were removed from all subsequently generated database searches. Full screening processes across each database are available upon request. For each of the database searches, one researcher (ES) performed a title and abstract screen of the potentially relevant papers (full-list available from the authors upon request) in order to identify descriptions or program evaluations that included medical students and focused on interprofessional training, community health settings, and innovation. This initial screen yielded 18 potentially relevant papers, which were then subjected to a full-text review by two researchers (ES and MR). Only five of these papers were sufficiently germane to the AoC program and are reviewed below (6–10).

### Program development and implementation

In 2013, the Harvard Medical School Center for Primary Care (the Center) launched the AoC program. The AoC program was developed through a two-pronged approach, which happened in parallel: 1) the literature review described above as well as a review of additional published and gray literature on training innovators, aimed to identify similar programs and generate possible ideas for program structure and content and 2) the Center's Inaugural Student Leadership Committee aimed to build an interprofessional community of students passionate about primary care with an impact beyond the walls of the Harvard Medical School. The Student Leadership Committee was also inspired by other innovations, entrepreneurship and design challenge grant programs both at Harvard University and at universities throughout the country. The Student Leadership Committee worked with the Center leadership and a faculty advisor from a CHC to develop a meaningful program that allowed them to work in multi-disciplinary teams and work with underserved communities.

The AoC program was divided into four phases: 1) achieving CHC buy-in and issuing a request for proposals (RFPs), 2) forming interprofessional teams, 3) implementing the systems-based innovation curriculum, and 4) program evaluation.

As a first step in building the AoC program, the Center assembled a management team that consisted of a faculty adviser, two student managers, and a program coordinator. The faculty adviser was recruited from a local CHC and was well known and highly regarded among Boston's CHCs, as she had held numerous leadership positions within a CHC. The faculty adviser's role consisted of liaising with the CHCs, advising the student managers on the day-to-day management of the AoC program, and making connections between the Center, AoC teams, and CHCs. The two student mangers were recruited from medicine and business. The student managers disseminated the RFP, tracked team's progress, identified resources needed for teams based on monthly feedback at curricular sessions, and collaborated on building the innovation curriculum. Finally, one of the program coordinators (PC) of the Center managed the administrative, financial, and logistical aspects of the program.

### Phase 1: community buy-in and identification of priority focus areas

The AoC program capitalized on the rich history of CHCs in the Boston area, which had one of the first CHCs (Columbia Point) in the nation, founded in 1965 as part of President Lyndon Johnson's War on Poverty. The core CHC mission has not changed through the years, as they are ‘designed to reduce or eliminate health disparities that affect racial and ethnic minority groups, the poor, and the uninsured’ (11). Today, nationwide, CHCs serve the primary healthcare needs of over 24 million patients in over 9,000 locations across the United States (12). CHCs are federally funded and committed to providing care for the uninsured regardless of ability to pay; CHCs do collect payment via the fee-for-service structure for insured patients (11). Given this rich history and the reach of CHCs, the AoC leadership team prioritized a challenge grant program that deeply involved CHCs.

The Center engaged with key CHC stakeholders through meetings with leaders of Greater Boston CHCs and the Massachusetts League of CHCs to test the idea and interest CHCs would have in collaborating on an innovation project with students. From these meetings, we obtained CHC buy-in and collaboratively identified the following key priority areas in need of innovative solutions: 1) patient access services, 2) mental health integration, 3) alternative visits (those that go beyond in-person doctor–patient consultation, such as group, home, or electronic visits), 4) information technology and social media, and 5) trainings for ancillary staff. Using these key priority areas, we created an RFP in the form of ‘challenge grants’. The Center updated the RFP's key priority areas in consultation with CHCs for the year 2 application process.

### Phase 2: formation of interprofessional teams

The RFP requested that students form interprofessional teams comprising members across Harvard University graduate schools, Harvard-affiliated residency programs, and health professional schools in Boston to propose an innovative solution addressing one or more of the key priority areas. To facilitate interprofessional team building, we disseminated the RFP announcement widely across the various Harvard University graduate schools through contacting each school's health interest group (e.g., the Health Entrepreneurship Group at Harvard Business School and Health Policy Group at Harvard Kennedy School); we also circulated the RFP to four internal medicine and pediatrics Harvard-affiliated hospitals’ residency programs. We held a series of mixers that allowed students and medical residents ample opportunity to meet each other, interact, and form teams based on interest. CHC representatives attended a number of the mixers to meet with students and trainees, and engage with student teams on co-writing a proposal and implementing the innovation at their respective CHCs. Additionally, we used online forum boards (proboards.com) where students and trainees from across the Harvard community met virtually, posted comments, and chatted with each other. We held a series of proposal writing workshops 2 months after the initial mixer events to support students in the creation of a proposal. Proposals were evaluated on a set of criteria and scored via a weighted point system that preferred teams with multiple disciplines represented, ideas with scientific merit, and teams that had achieved buy-in from CHCs.

### Phase 3: implementation of the systems-based innovations curriculum

The implementation of the AoC program took a semi-structured approach. Teams came together for monthly meetings during which the systems-based innovation curriculum was taught. The teams also gave two formal presentations at the mid- and end-of-year meetings in which they provided progress updates, consolidated lessons learned, reported milestones achieved, and identified key outcome measures of success; key outcome measures of success included number of patients recruited or engaged, successful protocol development or implementation and additional money raised to fund project (see Table 1 for outcome measures). Additionally, members of the AoC management team conducted at least one CHC site visit with the student team and held quarterly check-in conference calls with faculty mentors at participating CHCs. Teams received administrative support staff from the Center and had access to various experts and resources within the university to help them plan and implement their innovation.

**Table 1:** AoC project descriptions and main outcomes cohorts 1 and 2

Project title/cohort	Student disciplines	Main outcomes	CHC
Innovative Weight Loss Support via Mobile Technology and Social Networks (Y1/Y2)	Business, Medicine, Engineering	Incorporated into an LLC (HealtheTrek) Developed a beta application for testing on 120 users Received $40,000 continuation funding from AoC to refine and develop product Received Letter of Interest from Blue Cross/Blue Shield Innovation Fund	Union Square Family Health, Somerville, MA
Integrating Oral Health into Diabetes Group Visit Models (Y1)	Dentistry, Medicine	Created a standard operating procedure for integration of oral health and primary care for diabetic patients Introduced 80 diabetic patients to first-time dental care	Windsor Street Health Center, Cambridge, MA
Reducing Barriers to Care: Reproductive Health Group Visits at Malden High School (Y1/Y2)	Education, Medicine, Public Health	Started high school-based health services for the first time since 1986 with 50 students screened and counseled on STDs in Y1 Received $40,000 in continuation funding from AoC in order to scale up services Received funding from City of Malden to establish permanent school-based clinic Obtained part-time staffing of clinic by Tufts Family Medicine residents and attending	Malden Family Health Center, Malden, MA
Novel Educational Game for Improving Adherence in Pediatric Asthma Patients (Y1)	Business, Medicine, Public Health	Developed beta type of a game to improve adherence in pediatric asthma patients Due to the amount of time to code and create a virtual game, this product was not tested by the end of the grant year	Dimock Community Health Center, Roxbury, MA
Tai Chi for Improved Balance and Wellness among the elderly (Y2)	Business, Public Health, Medicine	Developed a protocol and tool kit for starting Tai Chi classes at CHCs with instructional videos 43 Tai Chi classes held, with an average of 10 participants per class	Bowdin Street Health Center, Dorchester, MA
Trial of new clinical position: Adolescent Health Coach (Y2)	Medicine, Public Health, Pediatrics Resident, Physician Assistant, Policy	54 youths screened and added to the health coach caseload Standard operating procedures developed for integration of adolescent health coach into CHC Position of adolescent health coach became a permanent position funded by clinic budget	Chelsea Health Center, Chelsea, MA
Closing the Loop between pharmacy and clinicians (Y2)	Medicine, Public Health, Business	Project had not impacted clinic by the end of the project year	Joseph M Smith Health Center, Boston, MA
Refer Smarter to decrease wait time for specialist visits (Y2)	Medicine, Business	Five physicians at CHC adapted application for referral 24 cases were referred though the application	Codman Square Health Center, Dorchester, MA

The systems-based innovation curriculum formed the core of the AoC program (see Table 2 for details of the innovation curriculum). The curriculum was designed to build skills in three particular areas: working with interprofessional teams, understanding Lean Start-up methodology, and peer mentoring through reflection and problem-solving. All AoC teams included participants from at least two disciplines. The Center engaged a healthcare strategy and innovation consultant to build a curriculum that taught students effective communication skills, including how to communicate across disciplines, set team norms and expectations, and resolve conflicts. Stalmeijer et al. (10) emphasize in their findings that interprofessional training in medical school benefits students as they progress in their careers, and how team member diversity plays a role in team conflicts. Additionally, skills in project management, task prioritization, budgeting, and work planning were also included in the curriculum to give participants skills to implement their innovation. The curriculum was also flexible enough to meet team's needs, as identified via monthly feedback forms and we were able to add relevant speakers (i.e., legal counsel, institutional review board) to the monthly seminars as requested by student teams.

**Table 2:** Systems-based innovation curriculum

Grant month	Topic	Description
1	Introduction to CHCs	Introduction to CHCs, their history of formation, their role in the community, populations typically served, how CHCs fit into the Boston area healthcare system
2	Introduction to Community Based Participatory Research (CBPR) and Collaboration	Introduction into principles of CBPR, forming collaborations with CHCs and community members; IRB considerations and process
3	Project Management I: Budgets, Work-Plans.	How to create, manage, and track budgets and work-plans, including online tools and resources
3	The Iterative Process: ‘Build–Measure–Learn’	Introduction to ‘Lean Start-up’ methodology, including how to quickly deploy beta versions, field test, and iterate
4	Project Management II: Communication Skills, Intellectual Property & Legal Issues	Developing effective communication plans and strategies with team members, CHCs and clients; introduction to intellectual property, content sharing, agreements between developers; how to incorporate and risks and benefits (non-profit vs. for-profit), resources for legal advice
5	Monitoring and Evaluation	Developing and tracking project indicators, markers of success, and reporting on indicators
5	Re-Iterating: Challenges and Opportunities	Using data collected from end users and other analytics to re-iterate products
6	No Seminar – Summer Month	
7	No Seminar – Summer Month	
8	Formal Mid-Year Presentations of Progress	
9	Data Management and Analysis	How to safely and securely store data; data analysis software; resources at Harvard for data analysis
10	Marketing and Scaling-up	Developing a brand for the product; developing pitches to donors/investors; developing scale-up plan
11	Developing Sustainability Plans	How to create long-term/sustainability plan; finding opportunities for future funding (private vs. public)
12	Final Showcase	

The second component focused on teaching students to use the Lean Start-up methodology of ‘build–measure–learn’ with continuous innovation, deployment, and revision (13). Teams were introduced to the Lean Start-up methodology through didactics, and directly implemented it through the creation of their innovation with the CHCs. This ‘loop process’ challenged student teams and their CHC partners to continuously test and deploy their ‘minimum viable product’ rather than waiting months/years to implement a ‘final’ polished product. Seeking continuous feedback from users/participants along the way allowed teams to generate and respond to data in a matter of days and weeks rather than waiting for the months or years required of traditional randomized clinical trials (13). This approach allowed for the testing of critical assumptions quickly and in context (14).

The third component of the curriculum consisted of critical reflection and experience sharing, which occurred during the monthly meetings. Teams reflected and shared their project's opportunities and challenges, allowing for time to incorporate new insights (15, 16) and help each other trouble shoot. Teams were expected to respond in real-time to feedback, create successive iterations and constantly refine their innovation with the goal of building a final product that is competitive for future seed or venture funding, or is adopted by the CHC or municipal departments of health.

### Phase 4: program evaluation

The Center for Primary Care evaluation team designed separate semi-structured interview tools for the AoC students and the students’ mentors. The interviews with students assessed three broad areas related to the AoC program: 1) how the AoC program contributed to the student's learning, including skill development and understanding of innovation; 2) the student's perspective on the year-long project and work in interprofessional teams; 3) the student's future career plans, including interest in primary care and working with underserved populations. In the mentor interviews, mentors were asked about their involvement in the project, the perceived impact of the project, and the program's effect on linking academic medical centers with CHCs.

Each semi-structured interview lasted, on average, 45 to 60 min for the students, and 20 to 30 min for the mentors. For the first cohort, 11 out of 16 AoC students were interviewed – eight over the phone and five in person; four mentors were interviewed via phone and two were not available for comment. For the second cohort, 13 out of 17 AoC students were interviewed – six via phone and seven in person; the four mentors were interviewed via phone. We did not interview mentors for the two projects with continued funding in cohort 2, as the students were more independent during their second year of funding and mentors were less active. Cohort 2 also participated in a pre and post survey, which was designed based on findings from the first year evaluation. The survey assessed students’ perceptions of their own skills and competencies.

Interviews were recorded with permission, transcribed, and entered into NVivo10, a qualitative data analysis software program (QSR International, Cambridge, MA). A thematic qualitative analysis approach was used to classify and examine the data; the Center's evaluation team iteratively developed a set of codes, which were based on the interview template as well as emergent themes. Findings were developed through data triangulation of key informant interview.

## Results

### Literature review

Although our initial search identified more than 800 papers related to interprofessionalism and medical student training, remarkably few publications reported on programs sharing features of the AoC. Only 18 studies reported on programs that included broad interprofessional teams, experiential learning or innovation. For example, many programs included students from various health professions (e.g., nursing, pharmacy, public health), but we found none that explicitly recruited students in pre-professional training from outside health-related disciplines (e.g., law, engineering, policy, or business). For example, Andrus and Bennett (6) reported on an interprofessional team-based learning program that matched medical students with nursing and public health students to identify and implement health promotion programs around access to preventative services, smoking cessation, and cardiovascular disease prevention.

Among the papers that did report any interprofessional experiential training programs, the learning and projects were situated either in hospitals or classrooms. For example, Stalmeijer et al. (10) described a program in Germany and the Netherlands in which multi-disciplinary teams were responsible for the design, organization, and delivery of one multi-disciplinary course. Their report focused on team dynamics, how interprofessional training in medical school benefits students as they progress in their careers, and how team member diversity plays a role in team conflicts. While we did identify a small number of studies describing interprofessional learning in community settings, their programs targeted health communication, health promotion and the establishment of student clinical experiences, without emphasizing clinical innovation and systems change. For example, Andrus and Bennett described a multi-year experience in Rochester, which centered on community-based projects in health promotion, which was identified as a need by the community and therefore received community buy-in and support.

Our literature review yielded no reports of programs describing interprofessional experiential learning in community settings, with student participants from both within and outside health-related disciplines, targeting systems-based practice. None of the studies identified in our search explicitly articulated the goal of training students to be leaders in clinical innovation.

### Outcomes of the AoC

The goal of the AoC program was to promote interprofessional teaming, train innovators and stimulate interest in primary care and working with underserved populations. The goal of the student evaluation was to ascertain success of the AoC program including skills gained, experience working in teams and any changes in student's future career plans. In year 1, the AoC received a total of 12 applications representing eight disciplines (seven Harvard graduate schools and MIT Sloan School of Management). From these, four teams representing five disciplines consisting of a total of 16 students were selected to be in the inaugural cohort of the AoC. In year 2, the AoC received 22 applications representing 10 disciplines (eight Harvard graduate schools, MIT Sloan School of Management, the Massachusetts Institute for Health Professionals, and Harvard affiliated hospital residency programs). From these four teams representing six disciplines consisting of 20 students and residents were selected to make up the year 2 AoC cohort.

### Outcomes of AoC: measures of project and curriculum success

Student participants perceived that their projects had positive impacts on their patients, the CHCs in which they worked, and on their own learning (see Table 1 for a detailed list of project outcomes). Some projects achieved moderate success while some projects did not move too far along in the implementation phase because of issues that were not predicted at the start of the program. For example, the weight loss and pediatric asthma mobile app teams reported that outsourcing the mobile health application software development portion of their projects was particularly difficult. However, they reported that their projects inspired conversations among the CHC staff about the possibilities of improving health through technology.

In both cohorts, teams struggled with the same challenges, namely schedule conflicts, establishing team roles, communication (both within teams and between teams and the clinics/mentors), and reaching consensus regarding the vision and goals of the innovation projects. Many of these reported student challenges were reflected in the improvements that CHC mentors suggested, which included:

- Clearly define both the student and CHC mentor's roles as soon as a cohort is selected. Some mentors also suggested that an initial planning meeting to define roles and clarify the partnership between the Center, student teams, and each community site would have been a valuable use of time.
- Manage students’ expectations around how they will be interacting and collaborating with the clinics. Along those lines, make sure students understand that they need to be accommodating of the clinic staff's time.
- Better plan for inconsistent student availability. Several mentors noted that one of the biggest challenges they faced was that students were in and out of the projects due to summer breaks, away rotations, and the start of a new school year. One mentor mentioned that increasing the team size and number of students might be a way to mitigate this issue, and to have students be more present and involved in the work.
- Protect the time of the CHC staff. Even though the compensation for CHC mentors was helpful, some CHCs noted that it was still challenging to protect provider time. The money provided did not match the revenues that the CHC would have generated if the clinician was seeing patients.

Students in both AoC cohorts reported that they developed the following professional skills as a result of their participation in the program:

- Communication with those outside their academic discipline
- Initiating change within a community organization
- Project management skills, specifically budgeting
- Research skills (e.g., leading focus groups and interviews, creating and conducting surveys, conducting a needs assessment)

The students believed that the curricular content of the monthly AoC sessions were relevant and effective. Students especially valued the sessions on project management and budgeting, navigating the IRB process, and learning to conduct focus groups and community-based research. Both cohorts of students appreciated the support and flexibility from the AoC program and staff. They reported that having access to their project mentors and the AoC program staff helped them think through their goals and objectives and work through the obstacles they encountered, but they also felt teams had the autonomy to make decisions and be creative. As one student reported:

I would say that the Agents of Change grant was a really unique blend of expertise and knowledge, the application of having student led learning. I think that everyone at the center was very available to help us negotiate issues, but they really just laid out the options that we had and the ways that we could move forward and then let us be the people to put that into effect or who moved to make these ideas happen.

As noted in the evaluation description, with the second cohort we implemented a pre- and post-evaluation survey. The post-evaluation survey indicated improvements in student preparation and knowledge. In the pre-evaluation survey, students, on average, ranked their preparedness to lead an innovation project to improve community health and to partner effectively with a CHC on improving community health 7 and 7.14 out of 10, respectively. In the post-evaluation survey students indicated ranks of 8 and 8.09. Additionally, student knowledge of barriers to accessing care and of challenges facing providers caring for underserved populations increased from ranks of 5.5 and 5.6 out of 10 to 7.18 and 7 out of 10, respectively. However, there were not any substantial changes in other categories, which included student feelings about primary care, motivations, goals, and desired resources.

### Outcomes of the AoC: students’ attitudes and perceptions

All of the students interviewed from both cohorts, with one exception, believed that the program successfully met its goal to empower students to find ways to improve healthcare delivery and solve problems facing vulnerable populations. In the first cohort, eight out of 11 students responded to the question about whether the program also promoted collaborative, multidisciplinary teams, of which six responded affirmatively; in comparison, 11 out of 13 interviewed students responded affirmatively to the question about whether the program promoted collaborative, multidisciplinary teams across Harvard's graduate programs.

In terms of the AoC's impact on students’ career plans, AoC students in both cohorts remarked that the experience gave them additional clinical experience, clarity on their career trajectories and opened the door to other professional opportunities. Furthermore, students reported that the AoC program exposed them to the realities of working in a community health setting, which they had learned about in the classroom setting. For the year 2 cohort, we also noted marked differences in the way students answered our open-ended question, ‘If you are not planning on practicing medicine, what are your ideal future career plans?’ The pre-survey reported answers such as managing a healthcare organization, pursuing careers in hospital administration, healthcare consulting, or global health delivery. By comparison, the post-survey demonstrated a shift in how students conceived their future careers, as students provided responses regarding pursuing potential opportunities in healthcare strategy (x2) or working in a healthcare start-up company or as an entrepreneur (x4).

## Discussion

The paradigm shift in healthcare education toward systems-based learning demands innovation in pedagogical strategies. The AoC represents an early experiment in experiential, interprofessional, and community-based learning, aimed at building trainees’ competencies in areas such as leadership, innovation, interprofessional team learning and care delivery at CHCs. Our literature search identified no programs similar enough to the AoC to serve as a precedent for the program. Our preliminary evaluation of the AoC suggested that its first two iterations generally achieved the goal of instilling the skills of working on an interprofessional team to develop systems-based solutions and innovations, and exposure to primary care delivery and complexities of CHCs. Additionally, our initial findings suggest that the program was not successful in increasing student interest in primary care.

We learned several important lessons from the implementation and evaluation of years 1 and 2 that informed improvements to the program's third cohort, four of which stand out and are discussed below.

### Lesson 1: improve alignment with CHC priorities

CHCs indicated their desire to provide input at the proposal phase to design a project aligned with their priorities. As such, the RFP requirements changed to mandate that student/trainee teams co-develop innovation proposals with CHCs, and require a letter of support from a partner CHC be submitted at the application stage.

### Lesson 2: rapid start-up phase

CHC mentors and AoC participants suggested adding an early ‘immersion experience’ for teams to rapidly start their project without interruption from school/hospital obligations, which were often cited as barriers to success in the evaluation of cohorts 1 and 2. A weekend retreat was introduced to frontload essential didactics from the systems-based innovation curriculum previously spread out over the first 4 months in order to give students a foundation and framework on which to build their project. Additionally, teams used the retreat to define roles, build communication plans, set team expectations and norms, and develop work plans, budgets, and timelines early in the life of the project.

### Lesson 3: expanding grant year from 12 to 18 months

A barrier to project success consistent across all projects was the loss of 3 to 4 project months in the summer when students were away from Boston or working at full-time internships. This resulted in only 8 to 9 months for project development and implementation. Extending the grant cycle to 18 months allowed more time for the teams and CHC to develop and test a minimally viable product and gather data on implementation.

### Lesson 4: performance based financing

A major change for cohort 3 was the introduction of performance-based financing in the form of tranche funding. For cohorts 1 and 2, teams were not required to detail a timeline for spending down their funding, and often, teams did not spend their entire $20,000 allocation. The AoC management team noticed that successful teams often spent more money than their counterparts. For cohort 3, teams were required to identify performance indicators for their project and submit to the AoC management team a spending plan linked to their identified performance indicators. Once indicators were met, additional money was allocated to the team. In this way, the AoC management team had a clear way to know if teams were meeting expectations; if targets were not being met, then the AoC management team could help address challenges and barriers faced by the team, or the team could be phased out of the AoC program, freeing up resources for other teams. At the time of manuscript submission, we have yet to evaluate this tranche funding approach.

Our evaluation of the AoC has several important limitations. The use of predominantly qualitative methods and reliance on participants’ retrospective account of the program and their projects makes the findings descriptive and potentially susceptible to social desirability biases. And while we tried in interviews to elicit an understanding of the projects’ impacts on the CHC and the broader community the CHC served, these questions returned very little useful data. In future evaluations, we aim to expand our sample size at the CHC level to include CHC staff members and patients involved with AoC projects. Finally, it is difficult to find a useful way of measuring participants’ understanding of the U.S. health system and its associated complexities.

## Conclusion

Based on the evaluation data from cohorts 1 and 2, and the enthusiasm of students, trainees and CHCs for the AoC program, we believe that this program is a replicable way to train students as innovators and members of interprofessional teams to address the current complex healthcare environment. Additionally, the AoC program effectively links academic medical centers with CHCs to promote health innovation for underserved populations, exposing students and medical trainees to the specific opportunities and challenges of CHCs and underserved populations.
